# Effects of probiotics on glycemic control and intestinal dominant flora in patients with type 2 diabetes mellitus: A protocol for systematic review and meta-analysis

**DOI:** 10.1097/MD.0000000000023039

**Published:** 2020-11-13

**Authors:** Yue Sun, Yucheng Huang, Fanghang Ye, Weiwei Liu, Xiaohua Jin, Kexin Lin, Jingjing Wang, Yongxiang Gao, Lisha He

**Affiliations:** a College of Basic Medicine; b College of Clinical Medicine; c College of International Education, Chengdu University of Traditional Chinese Medicine, Chengdu, P.R. China.

**Keywords:** intestinal dominant flora, probiotics, T2DM

## Abstract

**Background:**

With the rapid development of modern society, people's dietary structure has been changing accordingly. Diets high in salt, fat, and sugar have led to an increase in the incidence of diabetes year by year, posing a great threat to human health. More than 90% of diabetic patients have type 2 diabetes mellitus (T2DM). It is currently believed that the onset of T2DM is mainly related to factors such as genetics, insulin resistance, impaired insulin cell function, and obesity. The main mechanisms are as follows:

1. It affects the intestinal intake of single-chain fatty acid (SCFAs). The decrease of SCFAs leads to the decrease of the number of islet cells and the decrease of insulin sensitivity, which in turn leads to insulin resistance (IR).
2. Intestinal barrier function is inhibited, which leads to intestinal nonspecific inflammation. Then, it leads to changes in the structure and function of intestinal endothelial cells and affects the transport of insulin, which leads to IR.
3. Intestinal flora participates in bile acid metabolism and liver circulation, and affects the metabolism of carbohydrates and lipids. Imbalance of intestinal flora leads to disorders of carbohydrate and lipid metabolism.
4. Intestinal flora can reduce oxidative stress and improve islet cell function and insulin metabolism. Imbalance of intestinal flora can lead to impaired islet cell function and insulin resistance.
5. Normal intestinal flora can increase the content of YY peptide and correct the abnormal nerve conduction in hypothalamic feeding center.

The dominant flora of normal intestinal tract is mainly anaerobic bacteria which are beneficial to the human body. Under certain conditions, when intestinal flora is maladjusted, harmful bacteria and opportunistic bacteria become the dominant intestinal bacteria, resulting in metabolic disorders. Ingestion of probiotics can correct the imbalance of intestinal flora, and then, have a therapeutic effect on T2DM. Therefore, we designed this study to evaluate the effects of probiotics on blood glucose control and intestinal dominant flora in patients with T2DM.

**Methods:**

The retrieval period of meta-analysis literature is set from January 1, 1990 to September 2020. We will mainly search five English electronic databases, including Cochrane Library, Pubmed, Excerpt Medical Database (EMBASE), Science Direct and Web of Science, and search the following four Chinese databases: China Biomedical Literature Database (CBM), China National Knowledge Infrastructure (CNKI), China Science Journal Database (VIP), Wanfang Database, and so on. At the same time, the two reviewers will independently conduct research selection, data extraction and deviation risk assessment, and use Review Manager 5.3 software provided by the Cochrane Collaboration for meta-analysis and heterogeneity assessment.

**Results:**

This study will demonstrate an evidence-based review of probiotics on glycemic control and intestinal dominant flora in patients with type 2 diabetes mellitus.

**Conclusion:**

This study can be used to evaluate the efficacy and safety of probiotics on glycemic control and intestinal dominant flora in patients with T2DM.

**Registration number:**

is INPLASY202090104.

## 1 Introduction

There are more than a billion people with diabetes in the world,^[1]^ and more than 90% of them have T2DM.^[2]^ The prevalence rate of T2DM continues to increase, and it is generally recognized by the scientific community that the imbalance of intestinal flora is an important risk factor for T2DM.^[3]^

The structure and distribution of intestinal microflora in patients with T2DM are significantly different from those in normal subjects, which is due to the changes in the abundance of various microorganisms in the intestinal microecology. A comparative analysis of obese mice showed that the percentage of Lactobacillus in DM mice was higher than that in non-DM mice.^[4]^ In addition, the level of Bifidobacterium in intestinal microecology of normal population is higher.^[5]^ In contrast, the composition of butyrate-producing bacteria in T2DM, Proteus beta, Clostridium, Lactobacillus, and Haemophilus in the intestinal flora of T2DM patients is more different.^[6,7]^

The data show that through a variety of molecular mechanisms, intestinal flora imbalance affects T2DM insulin resistance-related metabolism and participates in insulin resistance-related signal transduction pathways.^[8]^ The function of intestinal barrier depends on bile acid, and the production and metabolism of bile acid requires the participation of intestinal flora. Bile acid signal transduction activates FXR and TGR5, which in turn activates various intracellular signal pathways. Under the action of intestinal flora, the bile acid signal was enhanced, and the changes of intestinal dominant flora weakened the activation of FRX and TGR5. Metabolic disorders are thus induced.^[9]^

In addition, SCFA is the fermentation product of intestinal flora. Some studies have shown that SCFA can improve insulin sensitivity by up-regulating protein kinase signals in muscle and liver. Moreover, SCFA can induce increased release of GLP-1 and PYY by binding to G protein coupled receptors.^[10]^ The diversity and high abundance of SCFA-producing strains in the intestine will improve the HbA1c level of T2DM patients. In turn, the imbalance of intestinal flora leads to the decrease of SCFA level, which will further lead to the occurrence of T2DM.^[11]^

On the other hand, the consequence of the imbalance of intestinal flora is the increase in the abundance of opportunistic pathogens. Proliferating Gram-negative bacteria produce and release more endotoxins. The increased endotoxin directly destroys the intestinal barrier and forms a complex with CD14 to activate the TLRs signal pathway. This process further leads to an increase in the level of pro-inflammatory cytokines, which in turn causes inflammation and increases oxidative stress.^[12]^ In addition, MAPK signaling pathway is activated, which inhibits the activation of insulin receptor substrate and induces insulin resistance and β-cell death.^[13]^

Probiotics can improve T2DM by improving intestinal flora, which have the potential value of intervention in T2DM. Through the supplement of probiotics to the animal model, some researchers found that probiotics effectively improved the blood glucose of T2DM animals.^[14]^ In addition, NF-κB can up-regulate pro-inflammatory cytokines and activate JAK/STAT3 signal pathway.^[15]^ Probiotics reducing proinflammatory cytokines by inhibiting NF-κB pathway is also proved to be an effective method for the treatment of diabetes. Inhibition of STAT3 expression can reduce blood glucose level and liver lipid accumulation, and increase liver glycogen.^[16]^ Some studies have shown that supplementation of probiotics can significantly improve the epithelial barrier and reduce the level of inflammatory cytokines, such as IL-8, TNF-α, and IL-β. Moreover, probiotics can regulate nutrient metabolism at the level of expressed genes, down-regulate the expression of GSK-3β, FAS and SREBP-1, and up-regulate Akt, thus improving T2DM.^[17]^ The regulation of probiotics on intestinal microecology can effectively avoid over harvesting of energy. In addition, oxidative stress plays an important role in the pathogenesis of T2DM. It was found that the supplementation of probiotics could significantly improve the level of FBS, TAS, GSH, and MDA, and the improvement of biomarkers of oxidative stress had a positive effect on maintaining glucose homeostasis.^[18]^ Probiotic supplementation has proved to be effective in controlling blood glucose homeostasis in patients with T2DM. At the same time, we speculate that probiotic supplementation can regulate intestinal microecology and actively improve intestinal dominant flora, which is beneficial to T2DM patients as a whole. Therefore, on the basis of evidence-based medicine, we intend to collect reports about the supplement of probiotics to control blood glucose in patients with T2DM and improve intestinal dominant flora in patients with T2DM, and conduct a systematic review and meta-analysis of its efficacy, so as to provide more high—quality clinical evidence for patients with T2DM.

## 2 Study aim

The goal of this study is to systematically evaluate the effects of probiotics on blood glucose control and intestinal dominant flora in patients with type 2 diabetes, and then analyze the safety and effectiveness of its efficacy.

## 3 Methods

### 3.1 Registration

The system evaluation programme has been registered in the “International Registration system Evaluation and Meta-Analysis Protocol platform” (INPLASY), the registration number is INPLASY202090104 (https://inplasy.com/inplasy-2020-9-0104/). This systematic review follows the guidelines of the Cochrane Manual for systematic Evaluation of interventions^[19]^ and the preferred reporting project of the systematic review and meta-analysis protocol (PRISMA-P).^[20]^ If there are any adjustments during this meta analysis, we will update the details in the INPLASY database. Because this study belongs to evidence-based medicine, it does not need medical ethical recognition.

### 3.2 Inclusion criteria

#### 3.2.1 Types of study

This study will only include randomized controlled trials (RCTs), non-RCTs, quasi-RCTs, reviews, case reports, and other types of studies will be excluded. And all relevant RCTs published in English and Chinese on probiotic preparation in the treatment of T2DM could be included.

#### 3.2.2 Participants or population

This review includes patients with T2DM, regardless of race, region, sex, and the phase of Diabetic complications and nosogenesis. Does not include other serious diseases, such as other heart, kidney, blood system diseases, severe hereditary diseases, etc.

#### 3.2.3 Intervention

The main intervention is to use probiotics and probiotics to treat T2DM. The intervention group will be treated with probiotics or probiotics alone, or combined with other conventional drugs on the basis of probiotics and their preparations for T2DM treatment. The control group was given other routine treatment, such as routine drug treatment, observation, nursing, and so on. The treatment time and period of treatment for the use of probiotics and their preparations are not limited.

#### 3.2.4 Outcomes

##### 3.2.4.1 Primary outcomes

The main purpose of this study was to observe the blood glucose control (fasting blood glucose, two-hour postprandial blood glucose, random blood glucose) in patients with T2DM under the intervention of probiotics, as well as the intervention of Clostridium lean, Clostridium globosum, Bacillus vulgaris, Bifidobacterium, and so on.

##### 3.2.4.2 Additional outcomes

Additional results included improvements in other intestinal floras and complications in patients with T2DM as indicators of secondary evaluation.

### 3.3 Search strategy

The retrieval period of meta-analysis literature is set from January 1, 1990 to September 2020. We will mainly search five English electronic databases, including Cochrane Library, Pubmed, Excerpt Medical Database (EMBASE), Science Direct and Web of Science, and search the following four Chinese databases: China Biomedical Literature Database (CBM), China National Knowledge Infrastructure (CNKI), China Science Journal Database (VIP), Wanfang Database, and so on. At the same time (HYC) searched the clinical trial registration and gray literature about probiotic treatment of T2DM in European Drug Administration (EMA) (www.ema.europa.eu/ema/), World Health Organization (WHO), International Clinical trial Registration platform (www.wh.int/ICTRP), and so on. The search strategy for the combination of subject words and free words is decided by all commentators. The key words for this study are: T2DM, probiotic preparation, probiotics, blood glucose, blood glucose control, fasting blood glucose, two-hour postprandial blood glucose, random blood glucose, Clostridium perfringens, Clostridium globosum, Bacillus vulgaris, Bifidobacterium, intestinal dominant flora, intestinal flora.

### 3.4 Study selection and management

**Figure 1:**
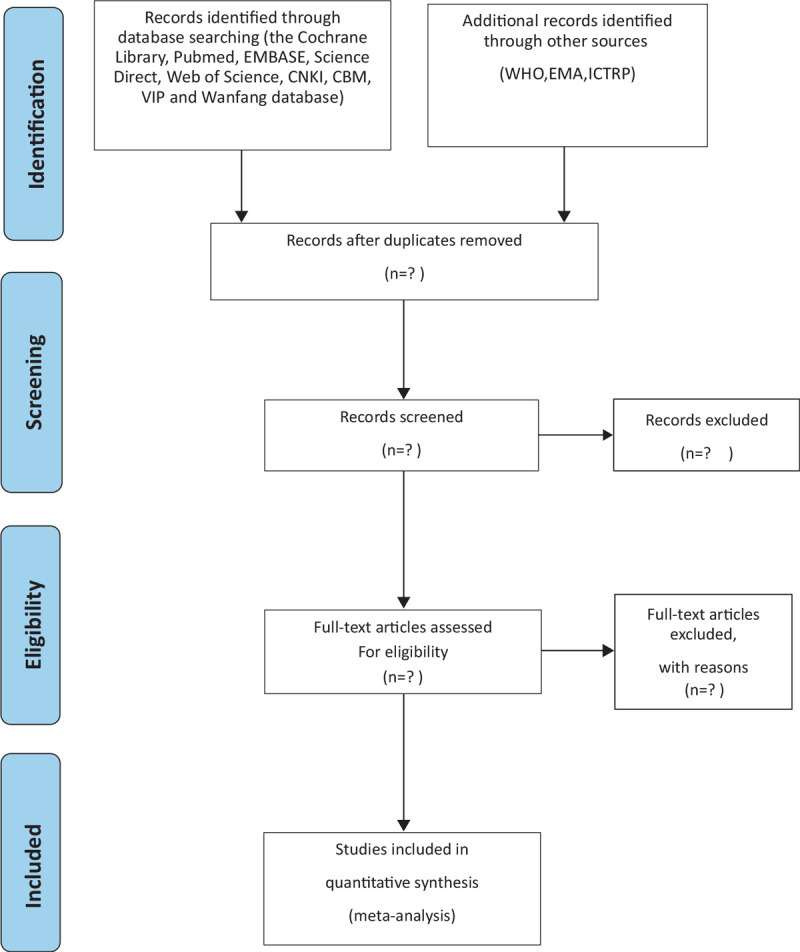
Flow diagram of the study selection process.

First, two reviewers (HYC and YFH) independently incorporated all the retrieved documents into EndNote X9. EndNote X9 software will be used for document management and the record search. Secondly, according to the pre-set inclusion and exclusion criteria, the two reviewers (HYC and YFH) independently consult the literature title and abstract, and finally eliminate the references that do not meet the requirements. Finally, two independent reviewers will carefully read the full text of each reference after preliminary screening and include references that meet the requirements of data analysis. If there are differences in the inclusion and exclusion of screening criteria, we will have a group discussion, the differences will be resolved by consensus, and the (SY) will be reviewed by third-party reviewers. The whole selection process will be represented by a prism flow chart (Fig. 1).

### 3.5 Data extraction and management

The two reviewers will extract data according to the Cochrane intervention system Review Manual and enter the EXCEL form. The table contains all the indicators that need to be analyzed in this study, from which the data can be refined. The two reviewers can independently fill in the primary and secondary result indicators (primary and secondary outcome indicators) that meet the requirements in this form. If there is a dispute, it can be discussed and agreed with third-party reviewers. The main data extracted from the EXCEL table are as follows:

comparison of published information (title, first author, year, national);
participants (age, sex, sample size, course of disease, type of T2DM and complications, inclusion and exclusion criteria, such as);
intervention (probiotics type, dosage, etc);
comparison (other forms of treatment, frequency, frequency of treatment).
results (primary and secondary outcome indicators [primary and secondary outcome indicators], evaluation time, follow-up, adverse events, etc). The above information will be independently verified by the two reviewers and the final decision will be made by the third-party reviewer (SY).

### 3.6 Assessment of risk of bias in included studies

The process of this study will be based on the deviation risk (ROB) assessment tool provided by the Cochrane manual to assess the quality of the included randomized controlled trials. The process will be assessed by two reviewers (HYC and YFH). Evaluation quality items include inclusion criteria, sample size estimates, baselines, randomization, allocation sequence hiding, binding, selective reporting, missing data management, and other deviations. According to the risk judgment criteria, we classify the quality of the above contents into three grades: “low deviation risk,” “high deviation risk,” and “unclear deviation risk.” If there are any other differences, we will discuss and reach an agreement with the third-party reviewer (SY).

### 3.7 Strategy of data synthesis

#### 3.7.1 Data analysis

We use Revman 5.3 software provided by Cochrane Collaboration for all statistical analysis and data synthesis. In this study, we will use a random effect model for meta-analysis. For binary data, 95% confidence interval (95%CI) and relative risk ratio (RR) is calculated. For continuous results, we will calculate the standard average deviation of 95% confidence interval, and the data results are represented by mean difference (MD) or standardized mean difference (SMD) and 95% confidence interval (95%CI). If there is statistical heterogeneity, the heterogeneity evaluation method is used.

#### 3.7.2 Assessment of heterogeneity

After chi-square test and *I*^2^ test, if the result is *I*^2^ > 50% *P* < .1, it indicates that the research result is heterogeneity, and random effect model should be used to analyze it.

### 3.8 Other publication bias analysis

#### 3.8.1 Subgroup analysis

In the case of heterogeneity of the data, the subgroup analysis was conducted according to the characteristics of the data (factors that may lead to heterogeneity), that is, the source of heterogeneity was discussed from the aspects of age, sex, regions, races, types of probiotic preparation, types of complications in patients with T2DM, and so on.

#### 3.8.2 Sensitivity analysis

When the effect of subgroup analysis is not satisfactory, we can conduct the sensitivity analysis to explore the impact of deviations in individual studies on the results. The main operation of sensitivity analysis is to eliminate the low-level quality research one by one, then merge the new data with Reven man5.3, and compare the new data with the previous results to judge the difference of sensitivity.

### 3.9 Grading the quality of evidence

In this study, reviewers will evaluate the quality of included randomized controlled trials. According to evidence quality, the intensity of the research evidence will be shown in the the “Grades of Recommendations Assessment, Development and Evaluation (GRADE)” standard,^[21]^ and the evidence quality evaluation of the main results will be divided into four grades: high, medium, low, and very low.

## 4 Discussion

There is a close relationship between intestinal flora and the pathogenesis of T2DM. More and more evidence shows that the therapeutic potential of probiotic intervention in T2DM is gradually being recognized based on the control of blood sugar in patients with T2DM and the improvement of intestinal dominant flora. The dominant intestinal flora mainly includes Clostridium lean, Clostridium globularis, Bacillus vulgaris, and Bifidobacterium. Too many intestinal flora products or structural components, with the increase of intestinal permeability, enter the circulatory system through the intestinal epithelium, which can promote the emergence of inflammatory reaction. LPS can initiate the release of proinflammatory cytokines and induce inflammatory response through the signaling transduction pathway of TLR.^[22]^ Secondly, SCFA is the fermentation product of intestinal flora. Studies have shown that the production of Th17 and Treg in different cytokine environments is regulated by SCFA, which induces the decrease of Th17 cells to reduce IL-17, and increase the level of Treg in the intestine to increase IL-10.^[23,24]^ In addition, SCFA acts on the NF-κB pathway and the corresponding TLR ligand, down-regulating the level of IL-8.^[25]^ Recently, some scholars have proposed that relying on NF-κB signal pathway can also reduce IL-18, IL-13, and IL-33.^[26]^ At the same time, SCFA activates STAT3 signal, and non-specific immune cells then regulate the immune response to cytokines and chemokines such as IL-6, IL-10, IL-22, and IL-23, which further antagonizes the inflammatory response.^[27]^ On the other hand, through the bile acid metabolism pathway, RORγ + Treg in the intestine is also controlled by intestinal flora to maintain host immune homeostasis.^[28]^ Inflammation will damage the structure and function of endothelial cells, lead to the imbalance of insulin transport, and then induce IR.^[29,30]^ In addition, by activating JNK, p38, and ERK accessory pathways, LPS inhibits the phosphorylation and expression of insulin receptor substrate through MAPK signal pathway, which is also one of the factors leading to IR. Therefore, the improvement and deterioration of T2DM patients are related to the changes of intestinal microflora. Probiotics can improve the intestinal microflora of patients with T2DM to some extent. There is evidence that probiotics combined with metformin can slow down insulin resistance in patients with T2DM by increasing the production of butyrate. Synbiotic probiotics are a commonly used intestinal microecological preparation in clinic.^[31]^ Synbiotics may affect the level of serum TNF-α and the expression of related MicroRNA in patients with T2DM.^[32]^

The level of intestinal inflammation in patients with T2DM is closely related to the homeostasis of intestinal flora in patients with T2DM. It is proved that intestinal flora in patients with T2DM is related to NF-κB signal pathway, tumor necrosis factor (TNF)-α, and SCFA. Therefore, the changes of intestinal dominant flora in patients with T2DM combined with a blood glucose index can be used as the main reference index to judge the diagnosis and prognosis of insulin resistance and intestinal inflammation in patients with T2DM. In recent years, a large number of clinical studies have shown that probiotics can effectively interfere with insulin resistance in patients with T2DM, but there is still a lack of evidence-based medical evidence to confirm whether probiotics and their probiotics play a positive role in the regulation of intestinal dominant flora and blood glucose control in patients with T2DM. In summary, we drafted this program to analyze and summarize the protective effects of probiotics on intestinal flora regulation and blood glucose control in patients with T2DM. This program will conduct meta-analysis for the existing clinical literature and provide clear evidence-based medicine for the clinical use of probiotics and their biological agents in the treatment of T2DM patients.

## Author contributions

**Conceptualization:** Yue Sun, Yucheng Huang.

**Data curation:** Yue Sun, Yucheng Huang, Fanghang Ye.

**Formal analysis:** Yue Sun, Fanghang Ye, Weiwei Liu.

**Funding acquisition:** Yongxiang Gao, Lisha He.

**Methodology:** Weiwei Liu, Lisha He.

**Project administration:** Yongxiang Gao, Lisha He.

**Resources:** Yue Sun, Kexin Lin.

**Software:** Yue Sun, Xiaohua Jin, Jingjing Wang.

**Supervision:** Yongxiang Gao.

**Writing – original draft:** Yue Sun, Fanghang Ye.

**Writing – review & editing:** Lisha He.
